# Diabetes knowledge and glycemic control among patients with type 2 diabetes in Bangladesh

**DOI:** 10.1186/s40064-015-1103-7

**Published:** 2015-06-20

**Authors:** Sheikh Mohammed Shariful Islam, Louis W Niessen, Jochen Seissler, Uta Ferrari, Tuhin Biswas, Anwar Islam, Andreas Lechner

**Affiliations:** Center for Control of Chronic Diseases (CCCD), International Center for Diarrhoeal Disease Research, Bangladesh (icddr,b), 68 Shaheed Tajuddin Ahmed Sarani, Mohakhali, Dhaka, 1212 Bangladesh; Center for International Health (CIH), Ludwig-Maximilians-Universität (LMU), Leopoldstraße 7, 80802 Munich, Germany; Centre for Applied Health Research and Delivery, Liverpool School of Tropical Medicine, Liverpool, L3 5QA UK; Diabetes Research Group, Medical Department 4, Ludwig-Maximilians-Universität (LMU), Ziemssenstr. 1, 80336 Munich, Germany; York University, Toronto, ON Canada; Clinical Cooperation Group Type 2 Diabetes, Helmholtz Zentrum München, German Research Center for Environmental Health, Neuherberg, Germany; German Center for Diabetes Research (DZD), Munich, Germany

**Keywords:** Diabetes knowledge, Perception, Risk factors, Management, Morbidities

## Abstract

**Aims:**

To explore the association between knowledge on diabetes and glycemic control among patients with type 2 diabetes in Bangladesh.

**Methods:**

A cross-sectional study was conducted among 515 patients with type 2 diabetes attending a tertiary hospital in Dhaka, Bangladesh. Trained interviewers were used to collect data on socioeconomic status, time since the onset of diabetes, co-morbidities, anthropometric measurements, blood tests, knowledge and perceptions about the causes, management, and complications of diabetes through face to face interviewers based on a structured questionnaire. Diabetes knowledge was reported using a composite score. Chi square tests and correlation analysis were performed to measure the association between knowledge on diabetes and glycemic control.

**Results:**

Overall, 45.6% participants had good, 37.7% moderate and 16.7% poor knowledge on diabetes. The mean composite score was 0.75 ± 0.28 and the proportion of participants with a score of ≤50% was 16.7%. Only 24.3% participants identified physical inactivity as a risk factor for diabetes. Knowledge on diabetes was significantly associated with education, gender, monthly income, duration of diabetes, body mass index, family history of diabetes, and marital status but not with glycated hemoglobin (HbA1c). Correlation matrix showed weak negative association between diabetes knowledge score and glycemic control (p < 0.001).

**Conclusion:**

Patients with type 2 diabetes in Bangladesh have limited knowledge on the causes, management and risk factors for diabetes, despite receiving professional health education and care in a tertiary diabetic hospital. Strategies to improve the quality of diabetes education and identifying other potential factors for glycemic control are important for ensuring optimum management of diabetes in Bangladesh.

## Background

Diabetes mellitus (DM) has emerged as a major public health challenge around the world. Low and middle income countries face the greatest burden of DM (Islam et al. 2014a, b, c, 2015). In 2011 the Diabetes Atlas of the International Diabetes Federation (IDF) estimated the global DM prevalence in the age group 20–79 years at 8.3%, which translates into 366.2 million people suffering from DM in 2011. The number of people living with DM is projected to reach 551.9 million by 2030 (Whiting et al. 2011). By 2030 Bangladesh is likely to emerge as the 8th highest ranking country in terms of the number of people with DM (Whiting et al. 2011).

Diabetes is a chronic condition, and diabetes-related complications like diabetic neuropathy, nephropathy, retinopathy, and diabetic foot ulcer are now alarming public health issues. These complications contribute to the decreased quality of life for affected individuals and their families, with a devastating long-term effect on their financial and social wellbeing. In a country like Bangladesh, where lack of health insurance forces individuals and families to bear the cost of health care, the financial impact of DM and its complications are much severe. Thus, DM ultimately affect the economy of the country as a whole though loss of productivity, morbidity and mortality (Islam et al. 2013). The management of DM largely depends on the affected person’s ability to pursue self-care in daily living. Patient education, therefore, is considered as an essential tool to control DM (Tan et al. 1997). Effective DM education, with consequent improvements in knowledge, attitudes and skills, leads to better control of the disease, and is widely accepted to be an integral part of comprehensive DM care and management (Assal et al. 1985; Norris et al. 2002; Asha et al. 2004). On the other hand, lack of knowledge and awareness may lead to increased susceptibility to the development of diabetic complications, and potentially higher healthcare costs among patients with DM.

Previous studies in Bangladesh have reported low level of knowledge on diabetes among the general population and especially among the newly diagnosed type 2 diabetes (T2D) patients (Saleh et al. 2012; Islam et al. 2014a, b, c, 2015). However, none of the studies explored the relationship between knowledge on diabetes with glycemic status. The objective of this study was to find out the association between knowledge on diabetes and glycemic control and overall perceptions about diabetes among patients with T2D in Bangladesh.

## Results

Among 515 patients in this study, 288 were females (55.9%). The mean ± SD age of the participants was 50.0 ± 10.1 years. An overwhelming majority of the respondents were married (87.18%) and had a family history of diabetes (68%). About 40% of the participants completed higher secondary education or above, while the median (IQR) income was 30,000 (34,000) Bangladeshi Taka (BDT) per month. The median (IQR) duration of DM was 3 (6) years. The socio-demographic and clinical characteristics of the patients has been published previously (Islam et al. 2014a, b, c, 2015).

Table 1 presents knowledge on diabetes of the participants in terms of different characteristics. Overall, 45.6% participants had good knowledge, 37.7% had moderate knowledge and 16.7% had poor knowledge on diabetes. Knowledge was better among males than females in all categories and this difference was statistically significant (P < 0.001). Knowledge was higher among those with higher education and the differences were statistically significant (P < 0.001). Knowledge was also significantly associated with monthly income (P = 0.02), marital status (P = 0.006), family history of diabetes (P < 0.001) and duration of diabetes (P = 0.001). However, there was no significant association between knowledge on diabetes and HbA1c status.

**Table 1:** Knowledge about diabetes by socio demographic and health characteristics

Variables	Good (235) %	Average (194) %	Poor (86) %	Total (515) %	P value
Sex					
Male	126 (53.6)	75 (38.7)	26 (30.2)	227 (44.1)	<0.000
Female	109 (46.4)	119 (61.3)	60 (69.8)	288 (55.9)	
Education					
No education	9 (3.8)	29 (14.9)	27 (31.4)	65 (12.6)	<0.000
Primary education	19 (8.1)	39 (20.1)	24 (27.9)	82 (15.9)	
Secondary education	67 (28.5)	64 (33.0)	28 (32.6)	159 (30.9)	
High secondary education	140 (59.6)	62 (32.0)	7 (8.1)	209 (40.6)	
Income (monthly) Bangladesh Taka (BDT)					
≤10,000 BDT	20 (8.7)	21 (11.3)	21 (25.3)	62 (12.4)	0.02
10,001–20,000 BDT	55 (23.9)	54 (29.0)	28 (33.7)	137 (27.5)	
20,001–30,000 BDT	52 (22.6)	28 (15.1)	14 (16.9)	94 (18.8)	
30,001–40,000 BDT	26 (11.3)	19 (10.2)	5 (6.0)	50 (10.0)	
40,001–50,000 BDT	33 (14.3)	24 (12.9)	6 (7.2)	63 (12.6)	
>50,000 BDT	44 (19.1)	40 (21.5)	9 (10.8)	93 (18.6)	
Marital status					
Married	211 (89.8)	172 (88.7)	66 (76.7)	449 (87.2)	0.006
Single	24 (10.2)	22 (11.3)	20 (23.3)	66 (12.8)	
Family history of diabetes					
Yes	187 (79.6)	115 (59.3)	48 (55.8)	350 (68.0)	<0.000
No	46 (19.6)	76 (39.2)	38 (44.2)	160 (31.1)	
HbA1c					
Optimal <7%	45 (32.6)	25 (26.9)	6 (17.6)	76 (28.7)	0.340
Fair 7–8%	37 (26.8)	26 (28.0)	8 (23.5)	71 (26.8)	
Poor >8%	56 (40.6)	42 (45.2)	20 (58.8)	118 (44.5)	
DM duration					
<5 years	123 (52.3)	133 (68.6)	59 (68.6)	315 (61.2)	0.001
≥5 years	112 (47.7)	61 (31.4)	27 (31.4)	200 (38.8)	

**Figure 1:**
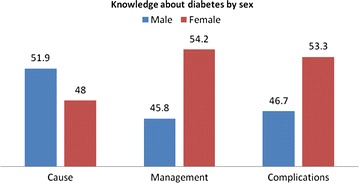
Knowledge about diabetes by sex.

Figure 1 shows knowledge on diabetes by gender. While a greater percentage of men had knowledge about the causes of diabetes, but women in general had better knowledge on the management and complications of diabetes.

The correlation matrix of diabetes knowledge score, duration of diabetes and HbA1c is presented in Table 2. There was a weak negative relationship between diabetes knowledge score and HbA1c which was statistically significant (p < 0.001). The relationship between diabetes knowledge score and diabetes duration was also weak (p < 0.05). Almost half of the participants (45.6%) had a composite knowledge score of 100, while 17% of them had composite knowledge score of 33 or less (Table 3).

**Table 2:** Correlation matrix for knowledge score, diabetes duration and HbA1c

	Score	HbA1C	DM duration
Score	1	−0.119	0.172^a^
HbA1c	−0.119	1	−0.123^b^
DM duration	0.172^a^	−0.123^b^	1

^a^Correlation is significant at the 0.01 level (2-tailed).

^b^Correlation is significant at the 0.05 level (2-tailed).

**Table 3:** Composite knowledge score of diabetes among study participants (by male/female and p value)

Composite score	Frequency (%)
n	515 (100)
0	23 (4.5)
33	63 (12.2)
67	194 (37.7)
100	235 (45.6)
Mean score ± SD	0.75 ± 0.28
Proportion of participants with score ≤50%	86 (16.7)

### Perceptions/beliefs about diabetes

Participants who answered “yes” to the question on knowledge about the causes, management and complications of diabetes were given an open-ended question to explain their position on various aspects of diabetes. Food habits, genetic predisposition, sedentary life style, lack of physical activity and obesity were the most commonly identified as causes of diabetes (Table 4). While more men than woman identified food habits as the cause of diabetes (p = 0.002), more women considered lack of physical activity as the most important cause (P < 0.001). A great majority of the participants believed that diabetes can be managed by diet and physical activity followed by medications, adhering to physician advice and maintaining a disciplined life. A higher proportion of men perceived that diabetes can be managed by changes in physical activity and medications than women, which was statistically significant (p = 0.007 and 0.006, respectively). The most common complications of diabetes as perceived by the participants were kidney problems and eye problems followed by heart diseases, blood pressure, bone and joint problems and other complications. More men perceived kidney and eye problems along with heart diseases as serious complications; while a statistically significant greater percentage of women considered bone and joint problems as serious complications (p = <0.05).

**Table 4:** Perceptions of diabetes among study participants

Perceptions	Male	Female	Total	p value
Cause				
Food habit	59 (25.99)	43 (14.93)	102 (19.80)	0.002
Genetic	50 (22.02)	46 (15.97)	96 (18.64)	0.08
Lack of physical activity	70 (30.75)	55 (19.09)	125 (24.26)	<0.001
Obesity	17 (7.50)	30 (10.40)	47 (9.10)	0.252
Medication	7 (3.08)	7 (2.43)	14 (2.71)	0.651
Blood sugar	7 (3.08)	3 (1.04)	10 (1.94)	0.095
Others	7 (3.08)	8 (2.77)	15 (2.91)	0.838
Management				
Diet	166 (73.1)	215 (74.65)	381 (73.79)	0.695
Physical activity	168 (74.00)	181 (62.84)	349 (67.76)	0.007
Medication	47 (20.70)	34 (11.80)	81 (15.72)	0.006
Follow physician advice	14 (6.16)	16 (5.55)	30 (5.82)	0.769
Discipline	4 (1.76)	1 (0.34)	5 (0.97)	0.104
Complications				
Kidney problems	99 (43.6)	100 (34.7)	199 (38.6)	0.04
Eye problems	87 (38.32)	86 (29.86)	173 (33.59)	0.043
Heart diseases	58 (25.55)	40 (13.88)	98 (19.02)	0.001
Blood pressure	10 (4.40)	12 (4.16)	22 (4.27)	0.894
Bone and joint problems	14 (6.16)	41 (14.23)	55 (10.67)	0.003
Liver problems	20 (8.81)	23 (7.98)	43 (8.34)	0.737
Neurological problems	15 (6.60)	19 (6.59)	34 (6.60)	0.996
Dermatology problems	11 (4.84)	18 (6.25)	29 (5.63)	0.493
Obesity	3 (1.32)	3 (1.04)	6 (1.16)	0.769
Others	109 (48.01)	111 (38.54)	220 (42.71)	0.031

## Discussion

This study, carried out in an urban tertiary care hospital in Bangladesh, explored the association between knowledge on diabetes and glycemic control. At the same time, it examined the knowledge and perception on diabetes among patients with T2D. Overall patients with T2D had limited knowledge about the risk factors, management and complications of diabetes. Less than half of the participants had good knowledge on diabetes with a mean composite score of 0.75 ± 0.28. Results of the study show that knowledge on diabetes is significantly associated with education, family history and duration of diabetes, but not with glycemic control.

Several studies have reported that knowledge about diabetes is generally poor among patients with diabetes in both the developed and developing countries (Wee et al. 2002; Al-Maskari et al. 2013; Deepa et al. 2014). According to the study by Saleh and colleagues among newly diagnosed T2D patients in Bangladesh, 16, 66, and 18% of respondents had good, average, and poor (GAP) basic knowledge on diabetes respectively while 10, 78, and 12% of respondents had GAP technical knowledge on DM (Saleh et al. 2012). However, in the current study the percentage of respondents with good knowledge on diabetes (45.6%) was much higher. It should be noted that our study was focused only on three domains of basic knowledge on the disease. Another study conducted among rural population in Bangladesh reported that only 50% of the participants identified physical inactivity was a risk factor for diabetes (Islam et al. 2014a, b, c, 2015). The study also found that males had generally more knowledge about diabetes. Moreover, following multivariate analysis the study found a significant association between level of education and knowledge on diabetes. However, none of these studies measured the association of knowledge on diabetes with glycemic status of the participants, which can provide evidence about the effectiveness of such interventions for diabetes management.

A study by Vishwanathan in India demonstrated that good knowledge on diabetes was higher among women and that half of the participants were unable to recognize the risk factors of the disease (Viswanathan et al. 2006). Another survey in a metropolitan city in India reported that about one third of the general public were unaware of the term ‘diabetes’ (Mohan et al. 2005). According to a study by Deepa and colleagues only 43.2% participants had heard about diabetes (58.4% in urban areas). The study also found that 63.4% of the population with diabetes were aware that DM could be prevented, while 72.7% of them knew that DM could affect other organs (Deepa et al. 2014). A study conducted in Singapore among the general population found that they have an acceptable level of knowledge on diabetes (Wee et al. 2002). On the other hand, a study conducted among a semi-urban Omani population found their level of knowledge on diabetes to be suboptimal (Al Shafaee et al. 2008). A study on people with diabetes attending the Aga Khan University Hospital in Karachi, Pakistan reported that 12, 35, and 53% of the patients, respectively had GAP knowledge of the symptoms, treatments, and complications of diabetes, respectively (Rafique et al. 2006).

The current study highlights certain beliefs and misconceptions prevalent among urban patients with T2D. Lack of knowledge might have an impact on their diabetes-related health practice. Traditional medicines are commonly used by many patients in Bangladesh for numerous chronic diseases. However, in the current study only 15.7% of the respondents thought that regular use of traditional medicine could cure diabetes. It is widely acknowledged that excessive sugar intake is a risk factor for DM (Johnson et al. 2007). However, although about 20% of the participants in this study identified food habits in general as a risk factor for diabetes, excessive sugar intake was not specifically identified as a risk factor. A great majority of the respondent in the study perceived that diabetes can be managed through changing lifestyle and diet.

Diabetes is one of the leading causes of end stage renal disease leading to kidney failure and a risk factor for myocardial infarction, stroke, retinopathy, and other serious diseases. Overall only 33.6 and 33.59% respondents in our study perceived that diabetes affects the eyes and kidneys, respectively. Although a small percentage of the participants could refer to dermatological problems as diabetes related complication, none of the participants could identify diabetes foot as such.

The study findings demonstrates that knowledge on the causes, management and complications of diabetes is limited in our study participants. In Bangladesh, where there is lack of access to healthcare for the general population, diabetes care hardly reaches those living in rural areas and hard-to-reach areas. Therefore, in these areas, knowledge on diabetes could be extremely low. Our study emphasizes the need for comprehensive diabetes education encompassing the risk factors, complications, management, diet, physical activity, life-style, self-management, and medication adherence among others. The findings also underscore the need for creating mass awareness and intensifying education measures for diabetes at large, including at risk populations, population at pre-diabetes phase and DM patients in particular along with identifying other factors that might affect glycemic control.

The study results show a weak negative relationship between knowledge on diabetes and glycemic control, which is similar to findings from other studies (Beeney and Dunn 1990; Coates and Boore 1996). This might be due to the fact that a large number of our patients were newly diagnosed cases of type 2 diabetes with a higher level of education. A previous study reported that patients achieved significantly higher target HbA1c rates than control subjects in the low literacy group, but not in the high literacy groups (Rothman et al. 2004). It is, therefore, likely that educational interventions targeted towards the specific population groups might work better to promote diabetes control (Berikai et al. 2007).

Our study had several limitations. First, data were limited to patients with T2D on oral medication attending an urban hospital in Dhaka city and thus does not represent the general population with diabetes in Bangladesh. Second, we could not use any valid questionnaire structure using Likert’s scale to score each item and measure the knowledge, as we could not identify any knowledge tool validated in our population. Also, we could not perform the validity and reliability of our knowledge tool. Third, as this is a descriptive study, factors or causes causing this potential association cannot be inferred. Fourth, we could not measure the motivation of the participants and treatment satisfaction, which might affect our findings. Finally, although several patients related and lifestyle factors are associated with glycemic control, we could not explore these association in our study.

## Conclusion

Patients with T2D in Bangladesh have limited knowledge on the causes, risk factors and management strategies of diabetes. The professional health education and care in a tertiary diabetic hospital do not appear to enhance knowledge on diabetes substantially. Our data also show that, there is a weak relationship between knowledge, duration of diabetes and glycemic control. Therefore, traditional diabetes education might not be sufficient to control diabetes. Innovative strategies should be identified and adopted to further to improve the quality of diabetes education to make it more effective. The study also underscores the need for further research to identify other critical factors enhancing glycemic control.

## Methods

### Study design and population

A cross-sectional study was carried out among 515 patients with T2D at the outpatient department (OPD) of the Bangladesh Institute of Health Sciences (BIHS) Hospital in Dhaka, Bangladesh between September 2013 to July 2014, as part of randomized controlled study on mobile phone intervention for diabetes in Bangladesh. The methods have been described in the study protocol elsewhere (Islam et al. 2014a, b, c, 2015). In short, the BIHS is a tertiary care hospital offering quality inpatient and outpatient care to people from all socioeconomic strata. As part of the registration process at BIHS hospital, each participant is required to attend a 30 min session with the BIHS health education officer for diabetes health education and nutrition counseling. A routine eye and dental examination is also carried out by an ophthalmologist and a dentist. All patients are attended by physicians who are certified diabetologists with several years of experience.

### Data collection process

A draft questionnaire was developed and modified following a well-designed pre-test. Selected participants were interviewed by trained interviewers to collect information about their knowledge on diabetes, socio demographic status. Anthropometric measurements were taken and biochemical tests for HbA1c were performed to gather glycemic status of the participants. Three qualified research assistants were involved in data collection under supervision of the research officer and the study physician.

### Ethical consideration

All participants were informed about the aims and objectives of the research and that they had the right to withdraw from the study at any point without any obligations. Written informed consent was taken from all participants before the interview. All data files were kept under lock and key and confidentiality of participants was ensured. The research protocol was approved by the Ethical Review Committee of the International Center for Diarrhoeal Diseases Research, Bangladesh (icddr,b).

### Variables and measurements

We developed a simple knowledge test to capture the basic knowledge of the patients in three dimensions of diabetes knowledge with both quantitative and qualitative components. The knowledge tool was developed by a team of investigators comprising of medical doctors, diabetologist, epidemiologist, health economist, and health systems specialist. We pretested the knowledge tool as part of the questionnaire at the outpatient department of another large hospital in Dhaka city (BIRDEM hospital) is a small sample of population (n = 50). Based on the pre-test, we modified the final questionnaire that would be easy for our participants to understand and answer. The three domains of knowledge on diabetes were: causes of diabetes, management of diabetes and complications of diabetes. A composite score of diabetes knowledge was developed as follow: For each “Yes” to a question, in any of these three domains (cause, management and complication of diabetes) a score of “1” was given while each “No” received a score of “0”. Thus, the least possible score was “0”, and the maximum possible score was “3” if all the three questions were answered. A “do not know” answer was not scored. We graded diabetes knowledge according to the following criteria: 3 = good, 2 = average and ≤1 = poor. A composite score in percentage was then derived by dividing each individuals score by the maximum score possible. For example, if an individual score is 2, then the composite score would be 2/3 × 100 = 66%. To better understand the perceptions on diabetes, participants were asked an open-ended question to describe what they knew about the three domains of diabetes: cause/risk factors, management and complications if they answered positive about that domain. All answers on perceptions were coded for data analysis, i.e. food habit, life style and so on.

### Data analysis

Data were presented as frequency and percentage for categorical variables and mean ± standard deviation (SD) for continuous variables. Chi square tests, t tests and Mann–Whitney U tests were performed to find out the difference between knowledge levels and other measures variables. Correlation matrix was developed to find out the association between knowledge levels and glycemic control. A *p* value of <0.05 was considered significant. Data analyses was performed using SPSS version 20 for Windows (IBM, NY, USA).
